# Quality Improvement Project (QIP) on Improving Awareness of Family Planning Risks With Valproate in Male Patients

**DOI:** 10.1192/bjo.2025.10339

**Published:** 2025-06-20

**Authors:** Ranjan Baruah, Shazlina Shahul, Mahmoud Odeh

**Affiliations:** Mersey Care NHS Foundation Trust, Liverpool, United Kingdom

## Abstract

**Aims:** A recent MHRA alert highlighted a possible association between valproate use by men around conception and an increased risk of neurodevelopmental disorders in their children.

To ensure that 100% of male patients on valproate in the practice are informed about family planning risks associated with valproate use and have documented advice recorded in patient electronic record system (EMIS) within 2 months.

**Methods:** The cohort consisted of 22 male patients on valproate, identified using the EMIS system. These patients were targeted for the intervention to ensure compliance with the MHRA guidance on family planning risks.

The first intervention involved sending an Accurx text message to all 22 patients. outlining the potential risks associated with valproate use around conception, need for effective contraception for both partners, and encouraged patients to contact the practice if they were planning to start a family.

After 1.5 months, a follow-up intervention was conducted. All 22 patients were contacted by phone to verify whether they had received the text, assess their awareness of the MHRA guidance, and provide family planning advice if they were previously unaware. Phone calls were made on two separate occasions, spaced two weeks apart, to maximise the likelihood of reaching patients.

**Results:** Of the 22 patients, 18 were successfully contacted. Amongst these, 8 confirmed receiving the original text message, while 10 didn’t. During the phone calls, it was noted that 5 patients were already aware of the MHRA alert, 13 were unaware but were informed of the guidance during the call.

Patients were also given an opportunity to ask any further questions or express concerns. For those who required additional information, the option of a consultation with the practice pharmacist was offered. Despite repeated attempts, 4 patients could not be reached.

**Conclusion:** This QIP revealed significant gaps in patient awareness of valproate family planning risks and the challenges of engaging patients with automated messaging. Key reflections include: Challenges with Automated Texting; Improved Communication with Phone Calls; Limited Patient Engagement.

This QIP successfully raised awareness of an MHRA alert regarding valproate use among male patients in a GP practice. While the initial response to automated texts was poor, follow-up phone calls ensured that most patients were informed. The project underscored the importance of a multi-modal, sustained approach to patient education for sensitive topics like family planning.

Recommendations

Routine Medication Reviews.

Pharmacist-Led Discussions.

Enhanced Communication Strategies.

Practice-Wide Alerts.

